# P373: Contamination and cross contamination on hospital surfaces (near touch site)

**DOI:** 10.1186/2047-2994-2-S1-P373

**Published:** 2013-06-20

**Authors:** D Lary, K Hardie, J Randle

**Affiliations:** 1 School of Molecular Medical Sciences, University of Nottingham, Nottingham, UK; 2 School of Nursing, Midwifery and Physiotherapy, University of Nottingham, Nottingham, UK

## Introduction

The hospital cleaning and it is link to healthcare associated infection is an on-going debate. There has been little evidence that environment cleaning is important in reducing spread of infection compared to other known risks. While a clean environment is usually taken for granted there is little evidence to show that cleanliness could be an important controlling factor in the spread of infection.

## Objectives

Investigate the contamination of environmental surfaces (near touch sites) and whether this plays a role in the transmission of HCAI and multi-drug resistant organisms and examine the role of cleaning in reducing the number of pathogens.

## Methods

Pathogens present on near touch sites were identified using molecular techniques and the role of cleaning surfaces was assessed by determining the total number of colony forming unit directly before and after cleaning, 2, 4, and 6 hour later. Antibiotic susceptibility profiling was applied to isolated bacteria and the genetic profiles of the isolates were evaluated using randomly amplified polymorphic DNA (RAPD) and the *spa*sequence-based typing methodfor discriminating between *S. aureus* isolates to evaluate the average linkage within samples.

## Results

Significant differences between surface contamination before and after cleaning (P<0.001) was observed. Similarly, the repopulation of bacteria ontouch surfaces was significantly lower just after cleaning and started to increase by time. A total of 509 near patient touch site swab samples were obtained, 21% were *S. aureus*, 14% *E. faecalis* and 11% *P. aeruginosa*. Samples collected showed resistance to commonly used clinical antibiotics with many being multi-drug resistant. In addition, samples collected on the same day, from different surfaces had similar microbial fingerprints and patterns of antibiotic sensitivity.

## Conclusion

Appropriate cleaning of surfaces decreased the amount of contamination and could ultimately play a role in decreasing the spread of infection. Moreover, from the microbial fingerprint and antibiotic sensitivity we suggest that hand contact with the surfaces initiates’ microbial transmission. Thus strict compliance of HCW activities with infection control procedures is vital to reduce the risk of cross infection from surfaces to susceptible patients.

## Disclosure of interest

None declared

